# Relationship Between Fear of Missing Out and Social Media Fatigue: Cross-Lagged Panel Design

**DOI:** 10.2196/75701

**Published:** 2025-07-18

**Authors:** Xue Yao, Junzhe Zhao, Wenfan Chao, Dongdong Gao, Minghui Wang, Guoxiang Zhao

**Affiliations:** 1 School of Psychology, Henan University, Jinming Road, Kaifeng, Henan, 475004, China, 1 368-378-6210

**Keywords:** college students, longitudinal design, negative cycle, social media fatigue, Fear of Missing Out scale, FoMO

## Abstract

**Background:**

In today’s digital landscape, social media proliferation offers easier access to others’ information and social activities but also introduces challenges such as social media fatigue (SMF). Previous studies have linked the fear of missing out (FoMO) to SMF; however, the directionality of this relationship remains unclear.

**Objective:**

This study aimed to explore the relationship between FoMO and SMF among college students and examine whether a mutually predictive relationship exists between them.

**Methods:**

This study adopted a longitudinal research design, administering questionnaires at two distinct time points (ie, T1 and T2) separated by a two-month interval. At T1, the questionnaire included demographic variables of the research subjects (student ID, name, gender, etc.), the Fear of Missing Out Scale, and the Social Media Fatigue Scale. At T2, the questionnaire consisted only of collecting demographic information (student ID and name) for matching, along with the same two scales. Following data collection, the datasets from the two time points were matched based on the demographic information; only successfully matched data were included in the final analyses. Subsequently, descriptive statistics and correlation analyses of FoMO and SMF at T1 and T2 were conducted using SPSS (version 26.0). Finally, a cross-lagged panel analysis was conducted using the FoMO and SMF at T1 and T2 to examine the autoregressive and cross-lagged relationships between the variables over time.

**Results:**

A total of 862 valid questionnaires were matched across the two data collection steps. Correlation analysis showed that FoMO at T1 was positively correlated with SMF at T1 (*r*=0.340; *P*<.001) and FoMO at T2 (*r*=0.332; *P*<.001) and SMF at T2 (*r*=0.229; *P*<.001). FoMO at T2 was positively correlated with SMF at T1 (*r*=0.217; *P*<.001) and T2 (*r*=0.417; *P*<.001). SMF at T1 and T2 were also positively correlated (*r*=0.425; *P*<.001). The cross-lagged regression results indicated that using the autoregressive path, FoMO at T1 positively predicted FoMO at T2 (*β*=0.300; *P*<.001), and SMF at T1 positively predicted SMF at T2 (*β*=0.351; *P*<.001). Additionally, FoMO at T1 positively predicted SMF at T2 (*β*=0.067; *P*=.003), and SMF at T1 positively predicted FoMO at T2 (*β*=0.156; *P*<.001).

**Conclusions:**

There is a bidirectional relationship between FoMO and SMF among college students, suggesting a mutual influence over each other and that this relationship perpetuates a negative cycle. These findings further extend existing research and provide insights for developing mental health programs for college students.

## Introduction

Do you feel exhausted after using social media? Is this different from when you first started using social media? While social media brings convenience to users’ life and work, it also introduces new psychological challenges. Over time, many users experience a shift in their engagement patterns, transitioning from active participation to network addiction, eventually culminating in social media fatigue (SMF). This transition is frequently accompanied by negative emotions such as anxiety and depression [1]. This phenomenon indicates that, following the explosive growth in social media usage, individuals gradually lose interest and begin to experience fatigue and anxiety, sometimes even developing a desire to disengage from these platforms [2]. These effects pose significant challenges not only to social media platforms but also to users’ mental well-being, and SMF is increasingly attracting scholars’ attention.

Social media fatigue refers to a negative emotional state subjectively experienced by the user, characterized by feelings of tiredness and boredom that arise from prolonged social media use [3,4]. Research has demonstrated that SMF may trigger negative emotions such as anxiety and depression among users [3]. Among employees, SMF has been linked to diminished self-regulation, potentially leading to unethical behaviors [5]. Among college students, SMF has been associated with a decline in academic performance [6-8]. Studies indicate that while individuals may initially engage in self-control and self-regulatory behaviors, many users do not proactively reduce inappropriate social media use in a timely manner [9]. Therefore, research on the factors that influence social media fatigue can help prevent individuals’ social media overuse and avoid further adverse outcomes.

One such factor is the fear of missing out (FoMO), a pervasive concern that others may be having rewarding experiences from which one is absent [10]. FoMO is particularly prevalent in the age of social media, where individuals often feel a strong desire to post and browse information through social media, and the desire for knowing other people’s (especially friends’) relevant content is particularly strong [11]. Studies show a strong positive correlation between FoMO and SMF [12,13], with FoMO effectively predicting SMF [14]. However, some studies suggest that FoMO may not directly predict SMF; instead, it influences SMF through compulsive social media use [3,15]. Despite these insights, the cross-sectional nature of previous studies limits the ability to establish causal relationships between FoMO and SMF, which also brings confusion and uncertainty to the development of intervention programs. Therefore, it is necessary to use longitudinal research to further clarify this problem and provide an empirical basis for the practice of mental health education.

On one hand, the theory of Compensatory Internet Use suggests that engaging in compensatory behaviors such as increased internet use helps regulate negative stress-related emotions like depression and anxiety [16]. Based on this theory, we hypothesize that FoMO can be alleviated through social media, but in the process, the intensity of social media use will also increase, resulting in fatigue among individuals. On the other hand, according to the Social Cognitive Theory of Mass Communication, social media use may enhance cognition, emotions, attitudes, and behaviors of users [17,18]. According to this theory, individuals experiencing SMF may reduce their social media use, only to later feel deprived of updates, thereby exacerbating FoMO [19]. Therefore, this study posits that FoMO and SMF may be causal to each other, and their interaction may lead to a negative feedback cycle.

This study aims to explore the causal relationship between FoMO and SMF, with the expectation of revealing the potential dynamic interaction between the two. Given the limitations of previous cross-sectional studies, this study employs a longitudinal design to explore the causal relationship between FoMO and SMF. By tracking social media usage, it more precisely captures the dynamic relationship between FoMO and SMF. Focusing on college students—a demographic heavily engaged with social media [20,21]—the core hypothesis of this study is that there is a mutual predictive relationship between FoMO and SMF among college students. Specifically, we propose that FoMO will intensify SMF, and SMF will further aggravate FoMO, thus forming a negative cycle. This cycle may have a series of negative impacts on college students’ mental health, academic performance, social relationships, and daily life. This study will use a longitudinal research design to analyze the interaction mechanism between FoMO and SMF. The study aims to clarify the causal relationship between FoMO and SMF in the time series, which will reveal the development of the negative cycle between the two. The study findings will provide a scientific basis for the formulation of subsequent intervention measures. These findings are intended to help college students better manage their social media usage behavior and reduce the negative impacts of FoMO and SMF. Ultimately, the study seeks to promote the mental health and all-round development of college students.

## Methods

### Study Design

This study used a longitudinal research paradigm with a two-month interval between data collection points. At the first time point (T1), participants were administered questionnaires that included sections for relevant demographic information, the Fear of Missing Out scale, and the Social Media Fatigue scale. At the second time point (T2), two months later, the same participants were administered questionnaires, which included sections containing the same scales and identification items for matching (ie, student ID and name).

### Ethical Considerations

The questionnaire and methodology for this study were approved by the Ethics Committee of Henan Province Key Laboratory of Psychology and Behavior (Ethics approval number: 20230918007). Using convenience sampling, questionnaires were distributed to students recruited from four universities in a central province of China. After obtaining consent and ensuring cooperation from the counselors or teachers, the researcher explained the purpose of research, measurement methods, questionnaire completion process, and important precautions to the participants. The questionnaires were then uniformly distributed and collected in the classrooms. To ensure the authenticity of the responses, participants were assured that the results would be used solely for academic research and would not impact them personally. Additionally, they were informed that they could withdraw from the survey at any time if they felt uncomfortable during the process. After matching the data, the student IDs and names of the participants were deleted before conducting the data analysis. Following data collection, small gifts were distributed to the participants.

### Sample Size

The sample size was estimated by using G*Power (version 3.1; Heinrich-Heine-University Düsseldorf). For an effect size, *f^2^*=0.15, a significance level, α=0.0,1 and power=0.95, the calculated minimum total sample size was 169. To account for potential sample attrition during the second phase of data collection, a conservative estimate of a 20% loss rate was applied, resulting in a revised minimum sample size requirement of 212. In the initial study, a total of 1,067 questionnaires were distributed, yielding 1,002 valid responses, resulting in an effective rate of 93.91%. In the second round, 964 questionnaires were distributed, with 899 valid responses, leading to an effective rate of 93.26%. A total of 862 sets of data were matched in the two data collections, and all the matched data were included in the final analysis. Missing data were minima,l and the few missing values were addressed using mean imputation.

### Measures

#### Fear of Missing Out Scale

The Fear of Missing Out scale, originally developed by Przybylski et al [10] and subsequently adapted by Li et al [22] to suit the Chinese context, consists of eight items and uses a 5-point Likert scale. The scale consists of two dimensions; all items are scored positively. The items are as follows: “I fear others have more rewarding experiences than me” and “When I have a good time, it is important for me to share the details online (eg, updating status).” The Cronbach α of the original questionnaires was 0.72 [22], while in this study, the Cronbach’α values of the two measures were 0.801 and 0.862 at T1 and T2, respectively.

#### Social Media Fatigue Scale

The Social Media Fatigue scale, initially developed by Lee et al [23], was translated and revised by Li et al [24] for use in the Chinese context. This scale consists of five items and uses a 5-point Likert scale. It is unidimensional, and all items are scored positively. One of the scale items is “I find it difficult to relax after continually using social media.” The Cronbach α of the original questionnaires was 0.91 [24], while in this study, the Cronbach α values of the two measures were 0.856 and 0.866, at T1 and T2, respectively.

### Data Analysis

SPSS (version 26.0; IBM Corp) was used to calculate the mean (SD) and Pearson’s correlation coefficients for FoMO and SMF at T1 and T2. Additionally, AMOS (version 24.0; IBM Corp) was used to construct the cross-lagged model to analyze the data. The model assessed autoregressive and cross-lagged coefficients, as well as the significance levels of path coefficients to establish the relationship between FoMO and SMF between T1 and T2.

## Results

### Summary Statistics

A total of 862 valid datasets were matched across two time points, and all were included in the analysis. Among the 862 matched datasets, participants’ mean ages ranged from 16 to 24 years, that is, mean 19.827 (SD 1.202). The sample included 521/862 sophomores (60.4%), 184 juniors (21.4%), and 157 seniors (18.2%). There were 294/862 (34.1%) students from urban areas and 568 (65.9%) from rural areas. The gender distribution included 391 (45.4%) men and 471 (54.6%) female students. Detailed results are shown in Table 1.

**Table 1.** Sociodemographic characteristics.

Categories and content	Datasets (N=862), n (%)
Place of Origin	
City	294 (34.1)
Rural	568 (65.9)
Gender	
Men	391 (45.4)
Women	471 (54.6)
Age	
16	2 (0.2)
17	3 (0.3)
18	64 (7.4)
19	347 (40.3)
20	211 (24.5)
21	149 (17.3)
22	66 (7.7)
23	18 (2.1)
24	2 (0.2)
Grade	
Sophomore	521 (60.4)
Junior	184 (21.4)
Senior	157 (18.2)

### Test of Common Method Biases

Harman’s one-factor test was conducted to assess common method bias [25]. An exploratory factor analysis was performed on all variables without rotation. The variance explained by the first factor was 26.71%, which is below the critical value of 40% [26], indicating that there was no serious common method bias in this study.

### Descriptive Statistics and Correlation Analysis

The results of the mean, standard deviation, and correlation analysis of the main variables in this study are presented in Table 2. This analysis revealed that FoMO and SMF exhibited a significant positive correlation at both time points and across time. Additionally, the initial levels of FoMO and SMF were significantly correlated with their respective levels two months later.

**Table 2.** Descriptive statistics and correlation analysis.

Variables and timepoints	Scores, mean (SD)	Correlation coefficients (*r*) at different timepoints			
		1	2	3	4
FoMO^a^ T1^b^	20.664 (6.000)	1			
FoMO T2^b^	20.910 (6.173)	0.332^d^	1		
SMF^c^ T1	14.071 (4.656)	0.340^d^	0.217^d^	1	
SMF T2	14.068 (4.173)	0.229^d^	0.417^d^	0.425^d^	1

a FoMO: Fear of missing out.

b T1, T2: First and second study timepoints.

c SMF: Social media fatigue.

d *P* < .001

### Cross-Lagged Panel Design

**Figure 1.**
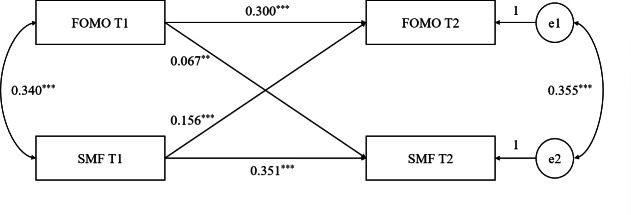
Cross-lagged model results. e1 and e2: error terms of FoMO and SMF at T2; FoMO: fear of missing out; SMF: social media fatigue; T1: first study time point; T2: second study time point.

A cross-lagged regression model was constructed using AMOS (version 24.0) to analyze the relationship between FoMO and SMF measured at the two time points (ie, T1 and T2). Findings indicated that the model was well-recognized, although the fitting index could not be output due to the saturation model. According to the test of significance of the model path coefficient, in the autoregressive path, FoMO at T1 positively predicted the FoMO at T2, and SMF at T1 positively predicted SMF at T2. In the cross-lagged regression paths, the FoMO in T1 positively predicted the SMF in T2, and SMF in T1 positively predicted the level of fear of missing out in T2 (see Table 3 and Figure 1).

**Table 3.** Cross-lagged analysis.

Path	Effect size	*P* value
FoMO^a^ T1→FoMO T2^b^	.300	<.001
SMF^c^ T1→SMF T2	.351	<.001
FoMO T1→SMF T2	.067	.003
SMF T1→FoMO T2	.156	<.001

a FoMO: Fear of missing out.

b T1, T2: First and second study timepoints.

c SMF: Social media fatigue.

## Discussion

### Principal Findings

Current studies have predominantly used cross-sectional designs to examine the relationship between FoMO and SMF, leaving the directional influence between these two inadequately addressed. To address this gap, this study used a longitudinal research design, surveying Chinese college students over a two-month interval. By using this approach, this study provided stronger evidence for the bidirectional influence between FoMO and SMF, thereby enhancing the understanding beyond prior cross-sectional findings [3,27]. Our findings suggest that interventions targeting either FoMO or SMF could effectively disrupt the negative cycle between them. These insights provide a clear direction for mental health education, emphasizing the importance of addressing both factors to promote healthier social media use and improve overall well-being among college students.

This study found a significant positive correlation between FoMO and SMF in both measurements among college students. Furthermore, the initial level of these constructs significantly predicted their development two months later, highlighting a cumulative risk effect over time. This suggests that if similar issues arise, timely intervention is crucial to prevent emotional problems. Therefore, it is essential to address both FoMO and SMF among college students. Proper guidance can help them understand and manage their FoMO, encouraging reasonable social media use.

Additionally, this study found an interactive relationship between FoMO and SMF. Specifically, the initial level of FoMO significantly predicted SMF two months later. Young people, who often experienced FoMO, tend to desire constant updates on others’ activities [10]. In today’s technologically advanced environment, social media serves as a crucial platform for accessing this dynamic information, fulfilling individuals’ need to establish and maintain relationships [28]. However, excessive engagement in social media driven by FoMO may lead to information overload and emotional exhaustion, thereby increasing the likelihood of developing SMF. This aligns with existing research results [14,29] and extends previous cross-sectional insights by demonstrating that fear of missing out not only has an immediate impact on SMF but also exerts a lasting influence over time.

Lastly, this study found that the initial SMF among college students can significantly predict the level of FoMO two months later, reinforcing the bidirectional nature of their relationship. One possible explanation is that SMF can lead to negative social media use behaviors, such as reducing the time or frequency of social media engagement. However, as other users continue to upload new content, individuals who temporarily reduce their social media use may feel they have missed more on news or opportunities when they return. This can exacerbate their FoMO, making them more eager to stay updated on others’ activities [30].

### Limitations

While the longitudinal design of this study mitigates some limitations inherent in cross-sectional research, this study has certain constraints. The study collected data at only two time points, which allowed for only a preliminary explanation of the directional relationship between FoMO and SMF. This approach does not fully capture the dynamic nature of the relationship between these two variables among college students. Future research should incorporate long-term and multifrequency follow-up studies to obtain a more comprehensive understanding of how this relationship unfolds over an extended period. In addition, this study only focused on the direct relationship between FoMO and SMF using a cross-lagged panel design, without considering potential mediating or moderating factors. Future studies should explore the underlying mechanisms and boundary conditions that influence this relationship, such as individual personality traits, coping strategies, and social media usage patterns.

### Conclusion

Based on the theory of Compensatory Internet Use and Social Cognitive Theory of Mass Communication, this study explored the relationship between FoMO and SMF among college students. Using a longitudinal design, the study conducted cross-lagged panel design on 862 matching data points collected over two months. These findings revealed a bidirectional influence between FoMO and SMF. Specifically, FoMO was found to positively and significantly predict SMF two months later, and vice versa, SMF also positively and significantly predicted FoMO levels two months later, suggesting a negative cycle. Additionally, the initial level of FoMO and SMF was a significant predictors of their respective development levels over the two-month period, indicating a cumulative effect. These results confirm a self-reinforcing negative cycle, emphasizing the need for interventions targeting both factors to promote healthier social media use and improve psychological well-being.

## Abbreviations

FoMO: fear of missing out
SMF: social media fatigue
